# *trans*-Dichloridobis[dicyclo­hex­yl(2,4,6-trimethyl­phen­yl)phosphane-κ*P*]palladium(II)

**DOI:** 10.1107/S1600536812040810

**Published:** 2012-10-06

**Authors:** Isaac Buthelezi, Haleden Chiririwa, Hezron Ogutu, Reinout Meijboom

**Affiliations:** a Research Centre for Synthesis and Catalysis, Department of Chemistry, University of Johannesburg, PO Box 524, Auckland Park, 2006, Johannesburg, South Africa

## Abstract

The title compound, [PdCl_2_(C_21_H_33_P)_2_], forms a monomeric complex with a *trans*-square-planar coordination geometry about the Pd^II^ atom which lies on an inversion centre. The Pd—P bond lengths are 2.3760 (13) Å, while the Pd—Cl bond lengths are 2.3172 (14) Å. The observed structure was found to be closely related to that of *trans*-dichloridobis[dicyclo­hex­yl(phen­yl)phosphane-κ*P*]palladium(II), [PdCl_2_{P(C_6_H_11_)_2_(C_6_H_5_)}_2_] [Burgoyne *et al.* (2012 ▶). *Acta Cryst*. E**68**, m404].

## Related literature

For a review on related compounds, see: Spessard & Miessler (1996 ▶). For the synthesis of the starting materials, see: Drew & Doyle (1990 ▶). For similar *R*—P_2_PdCl_2_ compounds, see: Ogutu & Meijboom (2011 ▶); Muller & Meijboom (2010*a* ▶,*b* ▶). For their applications, see: Bedford *et al.* (2004 ▶). For the closely related structure of *trans*-dichloridobis[dicyclo­hex­yl(phen­yl)phos­phane-κ*P*]palladium(II), see: Burgoyne *et al.* (2012 ▶). For isotypic structures, see: Clarke *et al.* (2003 ▶); Grushin *et al.* (1994 ▶); Vuoti *et al.* (2008 ▶).
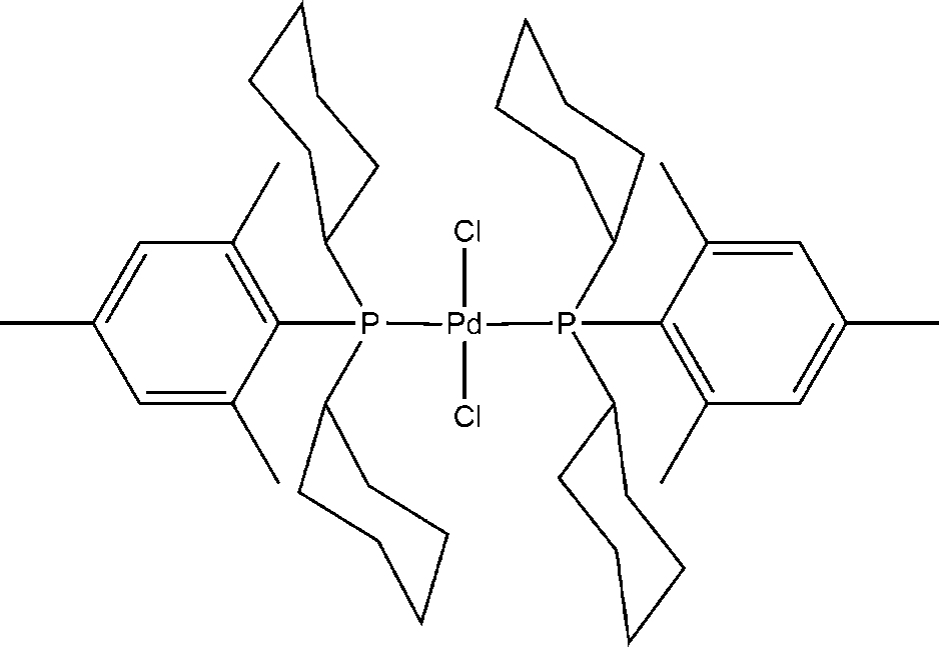


## Experimental

### 

#### Crystal data

[PdCl_2_(C_21_H_33_P)_2_]
*M* *_r_* = 810.19
Triclinic, 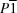

*a* = 9.466 (5) Å
*b* = 10.625 (5) Å
*c* = 11.527 (5) Å
α = 63.932 (5)°
β = 84.500 (5)°
γ = 75.874 (5)°
*V* = 1009.8 (8) Å^3^
*Z* = 1
Mo *K*α radiation
μ = 0.70 mm^−1^
*T* = 100 K
0.22 × 0.17 × 0.16 mm

#### Data collection

Bruker APEXII CCD diffractometer
Absorption correction: multi-scan (*SADABS*; Bruker, 2007) *T*_min_ = 0.861, *T*_max_ = 0.896
17391 measured reflections
4932 independent reflections
3385 reflections with *I* > 2σ(*I*)
*R*_int_ = 0.082

#### Refinement

*R*[*F* ^2^ > 2σ(*F* ^2^)] = 0.061
*wR*(*F* ^2^) = 0.165
*S* = 1.02
4932 reflections
217 parameters
H-atom parameters constrained
Δρ_max_ = 1.05 e Å^−3^
Δρ_min_ = −1.93 e Å^−3^

### 

Data collection: *APEX2* (Bruker, 2007 ▶); cell refinement: *SAINT-Plus* (Bruker, 2007 ▶); data reduction: *SAINT-Plus*; program(s) used to solve structure: *SHELXS97* (Sheldrick, 2008 ▶); program(s) used to refine structure: *SHELXL97* (Sheldrick, 2008 ▶); molecular graphics: *SHELXTL* (Sheldrick, 2008 ▶); software used to prepare material for publication: *SHELXTL*.

## Supplementary Material

Crystal structure: contains datablock(s) global, I. DOI: 10.1107/S1600536812040810/zj2095sup1.cif

Structure factors: contains datablock(s) I. DOI: 10.1107/S1600536812040810/zj2095Isup2.hkl

Additional supplementary materials:  crystallographic information; 3D view; checkCIF report

## Figures and Tables

**Fig. 1.**
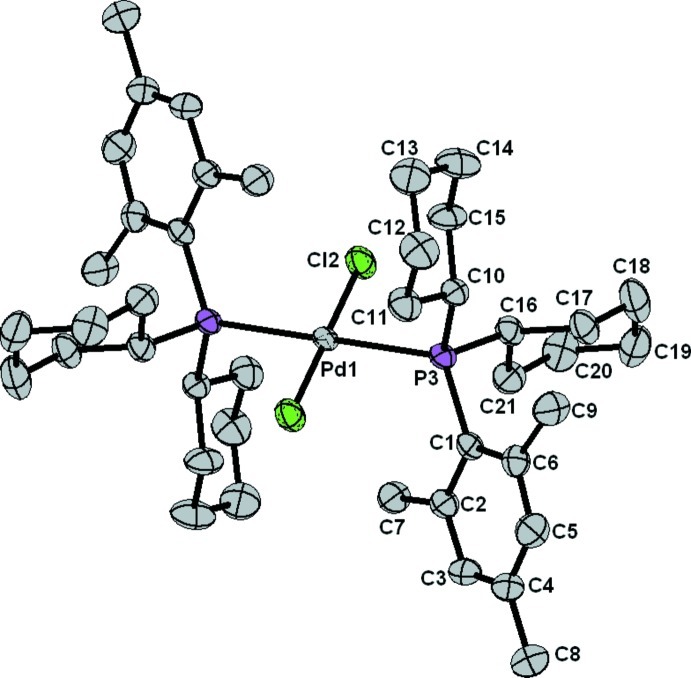
The structure of the trans-Dichloridobis[dicyclohexyl-(2,4,6 trimethyl phenyl)phosphane-jP] palladium(II) showing 50% probability displacement ellipsoids. Symmetry code to generate molecule through inversiont point: 1 - x, -y, 1 - z.

**Fig. 2.**
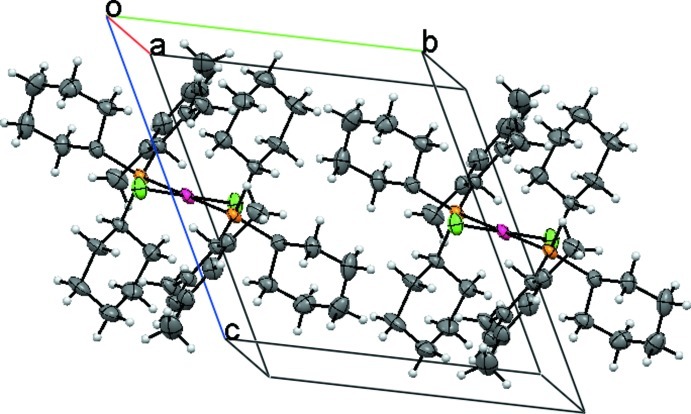
A perspective of trans-Dichloridobis[dicyclohexyl-(2,4,6 trimethyl phenyl)phosphane-jP] palladium(II) showing the molecular packing modes in the crystals.

## supplementary crystallographic information

### Comment

Complexes involving palladium metal centres are amongst some of the most popular catalytic precursors in organic synthesis due to their catalytic abilities. They are used in carbon-carbon bond formation reactions like the Heck, Stille and Suzuki reactions (Bedford *et al.*, 2004).[PdCl_2_(L)_2_] (L = tertiary phosphine, arsine or stibine) complexes can conveniently be prepared by the substitution of 1,5-cyclooctadiene (COD) from [PdCl_2_(COD)]. The title compound, *trans*-[PdCl_2_(C_21_H_33_P)_2_], crystallizes with the Pd atom on a center of symmetry and each pair of equivalent ligands in a mutually *trans* orientation.The geometry is,therefore, slightly distorted square planar and the Pd atom is not elevated out of the coordinating atom plane. All angles in the coordination polyhedron are close to the ideal value of 90°, with Cl2—Pd1—P1 = 88.39 (6)° and Cl2—Pd1—P1 = 91.61 (6)°. As required by the crystallographic symmetry, the Cl2—Pd1—Cl2 and P1—Pd1—P1 angles are 180°. The symmetry code used to define atoms through the inversion point is: 1 - x, -y, 1 - z.

The title compound compares well with other closely related PdII complexes from the literature containing two chloro and two tertiary phosphine ligands in a *trans* geometry (Muller & Meijboom, 2010a, b). The title compound, having a Pd1—Cl2 bond length of 2.3172 (14) Å and a Pd—P bond length of 2.3760 (13) Å, fits well into the typical range for complexes of this kind. Notably the title compound did not crystallize as a solvated complex; these type of PdII complexes have a tendency to crystallize as solvates (Ogutu & Meijboom, 2011).

Notably, the title compound is quintessentially isostructural with: [PdCl_2_{P(C_6_H_11_)_3_}_2_] (Grushin *et al.*, 1994); [PdBr_2_{P(C_6_H_11_)_3_}_2_] (Clarke *et al.*, 2003); and [PdCl_2_{P(C_6_H_11_)_2_(C_7_H_7_)}_2_] (Vuoti *et al.*, 2008) ((C_6_H_11_) = cyclohexyl, (C_7_H_7_) = *o*-tolyl). The Pd–P and Pd–X (X = Br and Cl) bond lengths were compared and it was observed that they were all within the same range of 2.3–2.4 Å. The angles between the bonds around the Pd atom were all observed to be approximately right angles.

### Experimental

Dicyclohexyl-(2,4,6 trimethyl phenyl)phosphine (0.11 g, 0.35 mmol) was dissolved in acetone (5cm^3^). A solution of [Pd(COD)Cl_2_] (0.05 g,0.17 mmol) in acetone (5 cm^3^) was added to the phosphine solution. The mixture was stirred for 5 minutes, after which the solution was left to crystallize. Yellow crystals of the title compound suitable for X-ray diffraction studies were obtained. ^1^H NMR (CDCl_3_, 400 MHz,p.p.m.): 6.9–6.8(*m*, 4H), 2.3 (*m*, 18H),1.5–1.4 (*m*, 16H),1.5 (*m*, 8H),1.6 (*m*, 16H),1.3 (*m*, 16H), 1.4 (*m*, 4H).^31^P NMR (CDCl_3_, 162.0 MHz, p.p.m.): 80.82. FTIR (cm^-1^):2920, 2850, 1713, 1678, 1597, 1553, 1442, 1337, 1074, 998, 883, 846, 728,

### Refinement

The aromatic, methine, and methyl H atoms were placed in geometrically idealized positions (C—H = 0.95–0.98) and constrained to ride on their parent atoms, with *U*_iso_(H) = 1.2*U*_eq_(C) for aromatic and methine H atoms, and *U*_iso_(H) = 1.5*U*_eq_(C) for methyl H atoms respectively. Methyl torsion angles were refined from electron density.

### Crystal data

**Table:**

C_42_H_66_Cl_2_P_2_Pd	*Z* = 1
*M**_r_* = 810.19	*F*(000) = 428
Triclinic, *P*1	*D*_x_ = 1.332 Mg m^−^^3^*D*_m_ = 1.332 Mg m^−^^3^*D*_m_ measured by not measured
Hall symbol: -P 1	Mo *K*α radiation, λ = 0.71069 Å
*a* = 9.466 (5) Å	Cell parameters from 3294 reflections
*b* = 10.625 (5) Å	θ = 2.2–28.3°
*c* = 11.527 (5) Å	µ = 0.70 mm^−^^1^
α = 63.932 (5)°	*T* = 100 K
β = 84.500 (5)°	Cube, yellow
γ = 75.874 (5)°	0.22 × 0.17 × 0.16 mm
*V* = 1009.8 (8) Å^3^	

### Data collection

**Table:**

Bruker APEXII CCD diffractometer	3385 reflections with *I* > 2σ(*I*)
Graphite monochromator	*R*_int_ = 0.082
φ and ω scans	θ_max_ = 28.3°, θ_min_ = 2.2°
Absorption correction: multi-scan (*SADABS*; Bruker, 2007)	*h* = −12→12
*T*_min_ = 0.861, *T*_max_ = 0.896	*k* = −14→14
17391 measured reflections	*l* = −15→15
4932 independent reflections	

### Refinement

**Table:**

Refinement on *F*^2^	Primary atom site location: structure-invariant direct methods
Least-squares matrix: full	Secondary atom site location: difference Fourier map
*R*[*F*^2^ > 2σ(*F*^2^)] = 0.061	Hydrogen site location: inferred from neighbouring sites
*wR*(*F*^2^) = 0.165	H-atom parameters constrained
*S* = 1.02	*w* = 1/[σ^2^(*F*_o_^2^) + (0.0919*P*)^2^] where *P* = (*F*_o_^2^ + 2*F*_c_^2^)/3
4932 reflections	(Δ/σ)_max_ < 0.001
217 parameters	Δρ_max_ = 1.05 e Å^−^^3^
0 restraints	Δρ_min_ = −1.93 e Å^−^^3^

### Special details

**Table:**

Geometry. All esds (except the esd in the dihedral angle between two l.s. planes) are estimated using the full covariance matrix. The cell esds are taken into account individually in the estimation of esds in distances, angles and torsion angles; correlations between esds in cell parameters are only used when they are defined by crystal symmetry. An approximate (isotropic) treatment of cell esds is used for estimating esds involving l.s. planes.
Refinement. Refinement of F^2^ against ALL reflections. The weighted R-factor wR and goodness of fit S are based on F^2^, conventional R-factors R are based on F, with F set to zero for negative F^2^. The threshold expression of F^2^ > 2sigma(F^2^) is used only for calculating R-factors(gt) etc. and is not relevant to the choice of reflections for refinement. R-factors based on F^2^ are statistically about twice as large as those based on F, and R- factors based on ALL data will be even larger.

### Fractional atomic coordinates and isotropic or equivalent isotropic displacement parameters (Å^2^)

**Table:**

	*x*	*y*	*z*	*U*_iso_*/*U*_eq_	
Pd1	0.5	0	0.5	0.02770 (17)	
Cl2	0.66664 (12)	0.13206 (13)	0.48974 (13)	0.0403 (3)	
P1	0.31238 (11)	0.14874 (12)	0.56557 (10)	0.0279 (3)	
C16	0.3797 (5)	0.2560 (5)	0.6307 (4)	0.0332 (10)	
C11	0.1511 (6)	0.2214 (6)	0.3425 (5)	0.0420 (12)	
H11A	0.0817	0.1659	0.4001	0.05*	
H11B	0.2285	0.1549	0.3197	0.05*	
C6	0.0268 (5)	0.1246 (5)	0.6872 (5)	0.0363 (11)	
C2	0.2357 (5)	−0.0711 (5)	0.7937 (5)	0.0358 (11)	
C1	0.1790 (5)	0.0641 (5)	0.6878 (4)	0.0316 (10)	
H1	0.1609	0.0137	0.6369	0.038*	
C4	−0.0041 (6)	−0.0812 (6)	0.8892 (5)	0.0438 (12)	
C21	0.4639 (5)	0.1621 (6)	0.7596 (5)	0.0405 (12)	
H21A	0.3976	0.1123	0.8265	0.049*	
H21B	0.5438	0.0881	0.7492	0.049*	
C17	0.2603 (5)	0.3749 (5)	0.6446 (5)	0.0415 (12)	
H17A	0.2087	0.4373	0.5608	0.05*	
H17B	0.1885	0.3314	0.7089	0.05*	
C15	0.3216 (5)	0.3781 (6)	0.3220 (5)	0.0435 (12)	
H15A	0.3587	0.4253	0.3663	0.052*	
H15B	0.406	0.3152	0.3018	0.052*	
C3	0.1420 (6)	−0.1368 (5)	0.8900 (5)	0.0427 (12)	
H3	0.1822	−0.2255	0.9604	0.051*	
C12	0.0713 (6)	0.3418 (6)	0.2195 (5)	0.0501 (14)	
H12A	0.028	0.2989	0.1747	0.06*	
H12B	−0.009	0.4051	0.2434	0.06*	
C7	0.3943 (6)	−0.1527 (6)	0.8096 (5)	0.0491 (14)	
H7A	0.4314	−0.173	0.894	0.074*	
H7B	0.4521	−0.0943	0.7411	0.074*	
H7C	0.4014	−0.2434	0.8039	0.074*	
C10	0.2180 (5)	0.2864 (5)	0.4122 (4)	0.0300 (9)	
H10	0.1369	0.3521	0.4343	0.036*	
C18	0.3265 (6)	0.4651 (6)	0.6878 (7)	0.0567 (16)	
H18A	0.3939	0.5129	0.6208	0.068*	
H18B	0.248	0.5408	0.6971	0.068*	
C20	0.5269 (6)	0.2537 (7)	0.8028 (6)	0.0527 (14)	
H20A	0.5996	0.2965	0.7393	0.063*	
H20B	0.5774	0.1917	0.8871	0.063*	
C9	−0.0548 (5)	0.2671 (6)	0.5890 (5)	0.0477 (13)	
H9A	−0.155	0.2882	0.6177	0.071*	
H9B	−0.056	0.2633	0.5057	0.071*	
H9C	−0.0064	0.3428	0.5796	0.071*	
C13	0.1717 (7)	0.4301 (6)	0.1292 (5)	0.0555 (15)	
H13A	0.1159	0.5087	0.0527	0.067*	
H13B	0.2475	0.3689	0.0991	0.067*	
C5	−0.0587 (5)	0.0501 (6)	0.7852 (5)	0.0449 (13)	
H5	−0.1604	0.0906	0.7816	0.054*	
C19	0.4088 (6)	0.3735 (7)	0.8154 (6)	0.0565 (15)	
H19A	0.3405	0.3313	0.8844	0.068*	
H19B	0.4537	0.434	0.8392	0.068*	
C14	0.2432 (6)	0.4931 (6)	0.1962 (5)	0.0517 (14)	
H14A	0.1681	0.564	0.2157	0.062*	
H14B	0.3144	0.5446	0.1373	0.062*	
C8	−0.1042 (7)	−0.1536 (7)	0.9946 (6)	0.0652 (18)	
H8A	−0.0615	−0.257	1.038	0.098*	
H8B	−0.199	−0.1378	0.9569	0.098*	
H8C	−0.1171	−0.1129	1.0575	0.098*	

### Atomic displacement parameters (Å^2^)

**Table:**

	*U*^11^	*U*^22^	*U*^33^	*U*^12^	*U*^13^	*U*^23^
Pd1	0.0255 (3)	0.0175 (3)	0.0286 (3)	0.00253 (18)	0.00010 (18)	−0.00339 (19)
Cl2	0.0323 (6)	0.0314 (6)	0.0560 (8)	−0.0062 (5)	0.0050 (5)	−0.0190 (6)
P1	0.0264 (6)	0.0194 (6)	0.0277 (6)	0.0018 (4)	−0.0007 (4)	−0.0043 (5)
C16	0.034 (2)	0.027 (2)	0.034 (2)	0.0008 (19)	−0.0038 (19)	−0.011 (2)
C11	0.042 (3)	0.035 (3)	0.041 (3)	−0.001 (2)	−0.007 (2)	−0.013 (2)
C6	0.032 (2)	0.032 (3)	0.042 (3)	−0.002 (2)	0.003 (2)	−0.017 (2)
C2	0.037 (2)	0.026 (2)	0.034 (2)	0.0014 (19)	0.0014 (19)	−0.007 (2)
C1	0.036 (2)	0.021 (2)	0.029 (2)	0.0017 (18)	−0.0018 (18)	−0.0067 (18)
C4	0.051 (3)	0.038 (3)	0.046 (3)	−0.021 (2)	0.016 (2)	−0.019 (2)
C21	0.038 (3)	0.037 (3)	0.035 (3)	0.002 (2)	−0.004 (2)	−0.009 (2)
C17	0.035 (2)	0.032 (3)	0.054 (3)	0.001 (2)	−0.002 (2)	−0.020 (2)
C15	0.042 (3)	0.035 (3)	0.031 (2)	−0.003 (2)	−0.003 (2)	0.004 (2)
C3	0.055 (3)	0.030 (3)	0.030 (2)	−0.007 (2)	0.008 (2)	−0.005 (2)
C12	0.051 (3)	0.049 (3)	0.044 (3)	−0.004 (3)	−0.016 (2)	−0.014 (3)
C7	0.049 (3)	0.030 (3)	0.040 (3)	0.013 (2)	0.005 (2)	−0.002 (2)
C10	0.027 (2)	0.024 (2)	0.027 (2)	0.0038 (17)	−0.0026 (17)	−0.0049 (18)
C18	0.046 (3)	0.044 (3)	0.088 (5)	−0.003 (3)	0.004 (3)	−0.040 (3)
C20	0.045 (3)	0.061 (4)	0.051 (3)	−0.001 (3)	−0.008 (3)	−0.028 (3)
C9	0.029 (2)	0.045 (3)	0.047 (3)	0.006 (2)	0.003 (2)	−0.009 (3)
C13	0.065 (4)	0.048 (3)	0.035 (3)	0.002 (3)	−0.013 (3)	−0.006 (3)
C5	0.029 (2)	0.047 (3)	0.057 (3)	−0.008 (2)	0.008 (2)	−0.023 (3)
C19	0.052 (3)	0.069 (4)	0.065 (4)	−0.016 (3)	0.015 (3)	−0.045 (4)
C14	0.051 (3)	0.039 (3)	0.038 (3)	−0.006 (3)	−0.012 (2)	0.007 (2)
C8	0.075 (4)	0.056 (4)	0.062 (4)	−0.024 (3)	0.032 (3)	−0.024 (3)

### Geometric parameters (Å, º)

**Table:**

Pd1—Cl2	2.3172 (14)	C15—H15A	0.99
Pd1—Cl2^i^	2.3172 (14)	C15—H15B	0.99
Pd1—P1	2.3760 (13)	C3—H3	0.95
Pd1—P1^i^	2.3760 (13)	C12—C13	1.502 (8)
P1—C10	1.859 (4)	C12—H12A	0.99
P1—C16	1.862 (5)	C12—H12B	0.99
P1—C1	1.868 (5)	C7—H7A	0.98
C16—C17	1.532 (6)	C7—H7B	0.98
C16—C21	1.541 (6)	C7—H7C	0.98
C11—C10	1.524 (7)	C10—H10	1
C11—C12	1.536 (7)	C18—C19	1.520 (9)
C11—H11A	0.99	C18—H18A	0.99
C11—H11B	0.99	C18—H18B	0.99
C6—C5	1.382 (7)	C20—C19	1.526 (8)
C6—C1	1.427 (6)	C20—H20A	0.99
C6—C9	1.503 (7)	C20—H20B	0.99
C2—C3	1.393 (6)	C9—H9A	0.98
C2—C1	1.431 (6)	C9—H9B	0.98
C2—C7	1.522 (6)	C9—H9C	0.98
C1—H1	1	C13—C14	1.509 (8)
C4—C3	1.364 (7)	C13—H13A	0.99
C4—C5	1.395 (8)	C13—H13B	0.99
C4—C8	1.510 (7)	C5—H5	0.95
C21—C20	1.522 (8)	C19—H19A	0.99
C21—H21A	0.99	C19—H19B	0.99
C21—H21B	0.99	C14—H14A	0.99
C17—C18	1.526 (7)	C14—H14B	0.99
C17—H17A	0.99	C8—H8A	0.98
C17—H17B	0.99	C8—H8B	0.98
C15—C14	1.534 (6)	C8—H8C	0.98
C15—C10	1.540 (6)		
			
Cl2—Pd1—Cl2^i^	180	C13—C12—H12B	109.2
Cl2—Pd1—P1	91.61 (6)	C11—C12—H12B	109.2
Cl2^i^—Pd1—P1	88.39 (6)	H12A—C12—H12B	107.9
Cl2—Pd1—P1^i^	88.39 (6)	C2—C7—H7A	109.5
Cl2^i^—Pd1—P1^i^	91.61 (6)	C2—C7—H7B	109.5
P1—Pd1—P1^i^	180	H7A—C7—H7B	109.5
C10—P1—C16	103.8 (2)	C2—C7—H7C	109.5
C10—P1—C1	110.9 (2)	H7A—C7—H7C	109.5
C16—P1—C1	104.3 (2)	H7B—C7—H7C	109.5
C10—P1—Pd1	104.11 (15)	C11—C10—C15	110.4 (4)
C16—P1—Pd1	114.10 (15)	C11—C10—P1	112.8 (3)
C1—P1—Pd1	118.79 (15)	C15—C10—P1	110.9 (3)
C17—C16—C21	109.7 (4)	C11—C10—H10	107.5
C17—C16—P1	113.7 (3)	C15—C10—H10	107.5
C21—C16—P1	112.9 (3)	P1—C10—H10	107.5
C10—C11—C12	109.6 (4)	C19—C18—C17	111.6 (5)
C10—C11—H11A	109.7	C19—C18—H18A	109.3
C12—C11—H11A	109.7	C17—C18—H18A	109.3
C10—C11—H11B	109.7	C19—C18—H18B	109.3
C12—C11—H11B	109.7	C17—C18—H18B	109.3
H11A—C11—H11B	108.2	H18A—C18—H18B	108
C5—C6—C1	119.4 (4)	C21—C20—C19	111.7 (5)
C5—C6—C9	114.2 (4)	C21—C20—H20A	109.3
C1—C6—C9	126.4 (4)	C19—C20—H20A	109.3
C3—C2—C1	119.4 (4)	C21—C20—H20B	109.3
C3—C2—C7	115.9 (4)	C19—C20—H20B	109.3
C1—C2—C7	124.7 (4)	H20A—C20—H20B	107.9
C6—C1—C2	117.3 (4)	C6—C9—H9A	109.5
C6—C1—P1	125.7 (3)	C6—C9—H9B	109.5
C2—C1—P1	116.9 (3)	H9A—C9—H9B	109.5
C6—C1—H1	90.7	C6—C9—H9C	109.5
C2—C1—H1	90.7	H9A—C9—H9C	109.5
P1—C1—H1	90.7	H9B—C9—H9C	109.5
C3—C4—C5	116.5 (4)	C12—C13—C14	110.5 (5)
C3—C4—C8	123.1 (5)	C12—C13—H13A	109.6
C5—C4—C8	120.4 (5)	C14—C13—H13A	109.6
C20—C21—C16	110.7 (4)	C12—C13—H13B	109.6
C20—C21—H21A	109.5	C14—C13—H13B	109.6
C16—C21—H21A	109.5	H13A—C13—H13B	108.1
C20—C21—H21B	109.5	C6—C5—C4	123.7 (5)
C16—C21—H21B	109.5	C6—C5—H5	118.2
H21A—C21—H21B	108.1	C4—C5—H5	118.2
C18—C17—C16	110.3 (4)	C18—C19—C20	109.5 (5)
C18—C17—H17A	109.6	C18—C19—H19A	109.8
C16—C17—H17A	109.6	C20—C19—H19A	109.8
C18—C17—H17B	109.6	C18—C19—H19B	109.8
C16—C17—H17B	109.6	C20—C19—H19B	109.8
H17A—C17—H17B	108.1	H19A—C19—H19B	108.2
C14—C15—C10	110.9 (4)	C13—C14—C15	112.5 (5)
C14—C15—H15A	109.4	C13—C14—H14A	109.1
C10—C15—H15A	109.4	C15—C14—H14A	109.1
C14—C15—H15B	109.4	C13—C14—H14B	109.1
C10—C15—H15B	109.4	C15—C14—H14B	109.1
H15A—C15—H15B	108	H14A—C14—H14B	107.8
C4—C3—C2	123.7 (5)	C4—C8—H8A	109.5
C4—C3—H3	118.1	C4—C8—H8B	109.5
C2—C3—H3	118.1	H8A—C8—H8B	109.5
C13—C12—C11	111.8 (5)	C4—C8—H8C	109.5
C13—C12—H12A	109.2	H8A—C8—H8C	109.5
C11—C12—H12A	109.2	H8B—C8—H8C	109.5

Symmetry code: (i) −*x*+1, −*y*, −*z*+1.
